# Ecological Predictors of Organelle Genome Evolution: Phylogenetic Correlations with Taxonomically Broad, Sparse, Unsystematized Data

**DOI:** 10.1093/sysbio/syae009

**Published:** 2024-04-05

**Authors:** Konstantinos Giannakis, Luke Richards, Iain G Johnston

**Affiliations:** Department of Mathematics, University of Bergen, Bergen 5007, Norway; School of Life Sciences, University of Warwick, Coventry CV4 7AL, UK; Department of Mathematics, University of Bergen, Bergen 5007, Norway; Computational Biology Unit, University of Bergen, Bergen 5006, Norway

**Keywords:** Comparative methods, ecology, mtDNA, organelle evolution, ptDNA, phylogenetic generalized linear model, phylogenetic linear model

## Abstract

Comparative analysis of variables across phylogenetically linked observations can reveal mechanisms and insights in evolutionary biology. As the taxonomic breadth of the sample of interest increases, challenges of data sparsity, poor phylogenetic resolution, and complicated evolutionary dynamics emerge. Here, we investigate a cross-eukaryotic question where all these problems exist: which organismal ecology features are correlated with gene retention in mitochondrial and chloroplast DNA (organelle DNA or oDNA). Through a wide palette of synthetic control studies, we first characterize the specificity and sensitivity of a collection of parametric and non-parametric phylogenetic comparative approaches to identify relationships in the face of such sparse and awkward datasets. This analysis is not directly focused on oDNA, and so provides generalizable insights into comparative approaches with challenging data. We then combine and curate ecological data coupled to oDNA genome information across eukaryotes, including a new semi-automated approach for gathering data on organismal traits from less systematized open-access resources including encyclopedia articles on species and taxa. The curation process also involved resolving several issues with existing datasets, including enforcing the clade-specificity of several ecological features and fixing incorrect annotations. Combining this unique dataset with our benchmarked comparative approaches, we confirm support for several known links between organismal ecology and organelle gene retention, identify several previously unidentified relationships constituting possible ecological contributors to oDNA genome evolution, and provide support for a recently hypothesized link between environmental demand and oDNA retention. We, with caution, discuss the implications of these findings for organelle evolution and of this pipeline for broad comparative analyses in other fields.

The statistical investigation of variables that are correlated across biological species can help reveal evolutionary and organismal mechanisms and relationships (Paradis 2014). In such investigation, controlling for the relatedness of species is essential to avoid false positive (FP) and negative (FN) results. For example, there are thousands of eukaryotic species which have both fur and four limbs. But these do not constitute thousands of independent samples supporting a correlation between these variables, because they inherited those properties from a common mammalian ancestor. A wealth of approaches exists for comparative analyses accounting for such phylogenetic structure. Early milestones in addressing these questions were Felsenstein’s independent contrasts approach (1985) and Grafen’s phylogenetic regression with generalized least squares (1989), originally developed with an explicit or implicit picture of continuously varying characters. More recently, the range of approaches has dramatically expanded to offer statistically powerful methods for specific circumstances (Paradis 2014). For questions of variables that do not follow the continuously varying picture, these include a development by Pagel for binary characters (1994), and phylogenetically embedded generalized linear models for different response variable structures, discussed and implemented, for example, in Ives and Garland (2014) and Tung Ho and Ané (2014) following work by (Paradis and Claude 2002; Ives and Garland 2010).

As with any statistical method, these approaches require some critical assessment, particularly when their underlying assumptions are challenged. Maddison and FitzJohn (2015) discuss how several well-principled approaches still suffer in practice from the problem of pseudoreplication, where identical values of a discrete trait can be observed within a clade because its constituent species share properties with a common ancestor, rather than because of independent realisations of a “generative” correlation. Uyeda et al. (2018) provide another deep survey of some shortcomings of phylogenetic comparative methods, highlighting that many comparative approaches are still challenged by singular evolutionary events and that alternative approaches exploring conditional dependencies between variables can be informative. Losos (2011) also draws attention to the limitations of phylogenetic comparative methods, noting that they are connected to observation patterns rather than generative processes, and the underlying phylogenies themselves are rarely free from uncertainty. Maddison (2000) proposes a different class of approach where phylogenetically independent sets of observations are identified and analyzed together; Maddison and FitzJohn (2015) discuss an approach where such independent subsets of the phylogeny are identified before being subjected to, for example, Pagel’s approach (1994). Careful treatments and commentaries emphasize that the statistical power and effect of correcting for phylogenetic correlations depend on the specifics of the problem (Rohle 2006; Revell 2010), and that detailed investigation of a method’s performance on synthetic data can make interpretation clearer. Two important common themes run through these discussions: the necessity of comparing summary statistics and hypothesis testing outcomes to simulations under null and alternative hypotheses; and the necessity of characterizing the influence of any assumption breaking or other challenges to method applicability (Revell 2010; Losos 2011; Uyeda et al. 2018).

Here, we investigate a particular evolutionary question where many common comparative assumptions are violated: what clade-specific features are linked to the retention of genes in organelle DNA (oDNA)? Across eukaryotes, gene content in mitochondrial DNA (mtDNA), and plastid DNA (ptDNA) have been dramatically reduced since the endosymbiotic events that created the organelles and now varies dramatically across species (Keeling 2010; Smith and Keeling 2015; Johnston and Williams 2016; Janouškovec et al. 2017; Roger et al. 2017; Johnston and Burgstaller 2019; Mohanta et al. 2020; Giannakis et al. 2022). The genes retained in oDNA are of central importance in bioenergetics and metabolism, with impact from human diseases (Taylor and Turnbull 2005; Wallace and Chalkia 2013; Stewart and Chinnery 2015) to crop production (Havey 2004; Mackenzie 2010; Chen and Liu 2014; Chustecki and Johnston 2024). The question of why particular *genes* are retained across species has been studied for some time: hypotheses include the favoring of genes encoding products that are hard to import to the organelle after expression outside it (von Heijne 1986; Björkholm et al. 2015), genes that allow direct local control of organelle function (Allen 2015; Allen and Martin 2016), are energetically favorable to retain (Kelly 2021), and many more, reviewed and quantitatively compared in (Johnston and Williams 2016; Giannakis et al. 2022). However, the dual question of why particular *species* retain a given number of genes has received less attention. Some specific hypotheses have been proposed: parasitic organisms are more free to lose or transfer control of their bioenergetic organelles (Hjort et al. 2010; Keeling 2010; Smith and Keeling 2015; Sanchez-Puerta et al. 2023), gene transfer is more beneficial in plants with self-fertilization and clonal modes of reproduction (Brandvain et al. 2007; Brandvain and Wade 2009). More generally, different energetic economics across taxa may help shape oDNA gene profiles (Kelly 2021), a “burst-upon-drift” population genetic process may contribute to clade-specific retention patterns (Butenko et al. 2024), different mutation rates across taxa may favor different balances between retention and transfer (Lynch et al. 2006), and the copy number of organelles per cell may limit the capacity for gene transfer (Barbrook et al. 2006). However, with these exceptions, the general reasons why different species retain different organelle gene counts remain open.

Recent modeling work has suggested that a species’ environment may be responsible for some of this diversity (García-Pascual et al. 2023). Specifically, the retention of more oDNA genes is predicted to be beneficial in organisms whose environments result in strong, oscillating metabolic or bioenergetic demands (e.g., diurnal or tidal oscillations). In such environments, oDNA gene retention ensures that individual organelles can respond to changing demands more rapidly and individually than if essential genes are transferred to the nucleus—following the colocation for redox regulation (CoRR) hypothesis (Allen 2015; Allen and Martin 2016). Organisms that exist in more stable, less demanding environments (or can move away from challenges) are predicted to have less need for rapid, local organelle responses, and thus retain fewer oDNA genes. This theory captures some broad observations: intracellular parasites typically retain very few oDNA genes (Hjort et al. 2010; Keeling 2010); fungi and motile metazoans (less exposed to, or capable of moving from, fluctuating environmental conditions) retain more; plants (subject to diurnal fluctuations) more still (Johnston 2019); and algae and other species in intertidal and estuarine conditions often retain more yet (Keeling 2010). We wanted to ask whether more specific information on species ecology and environments could help quantitatively support this hypothesis.

However, the nature of this question poses several challenges to a data-driven comparative analysis. In a sense, the simplest case for phylogenetic comparative work is two continuously varying characters, one predictor and one response, that are perfectly observed and evolve according to Brownian motion on a perfectly known (and balanced) phylogeny (Felsenstein 1985; Symonds and Blomberg 2014). Our system departs from this ideal in several ways:

- The predictor is an ecological factor (often binary) and the response is an ordinal (gene count);
- Evolution proceeds by monotonic decrease of the response variable, at a rate and to an extent that may depend on the predictor—and hence our predictor influences both the evolutionary process and the subsequent pattern of observations (Losos 2011);
- The branch lengths of the phylogeny are not known in general;
- Some clades are both highly overrepresented in the dataset and have very similar response values (e.g., metazoans, which form the majority of mtDNA samples and all have almost identical mtDNA gene profiles);
- The observations of the predictor on the phylogeny’s tips are sparse: a positive observation typically corresponds to a positive value, but a negative observation may correspond either to a negative value or to an absent observation, leading to biased observation error (Hansen and Bartoszek 2012).

We, therefore, proceed by establishing a set of synthetic control cases where the performance of comparative methods is tested on simulated systems reflecting these complications (Revell 2010). The insight from these controls, coupled with a curation of ecological and oDNA sequence data, allows us to establish and interpret the test cases: comparative analysis of oDNA gene counts and variables describing organismal ecology and environments across eukaryotes.

## Materials and Methods

### Simulating Organelle-Like Evolution and Data Sparsity

**Figure 1.**
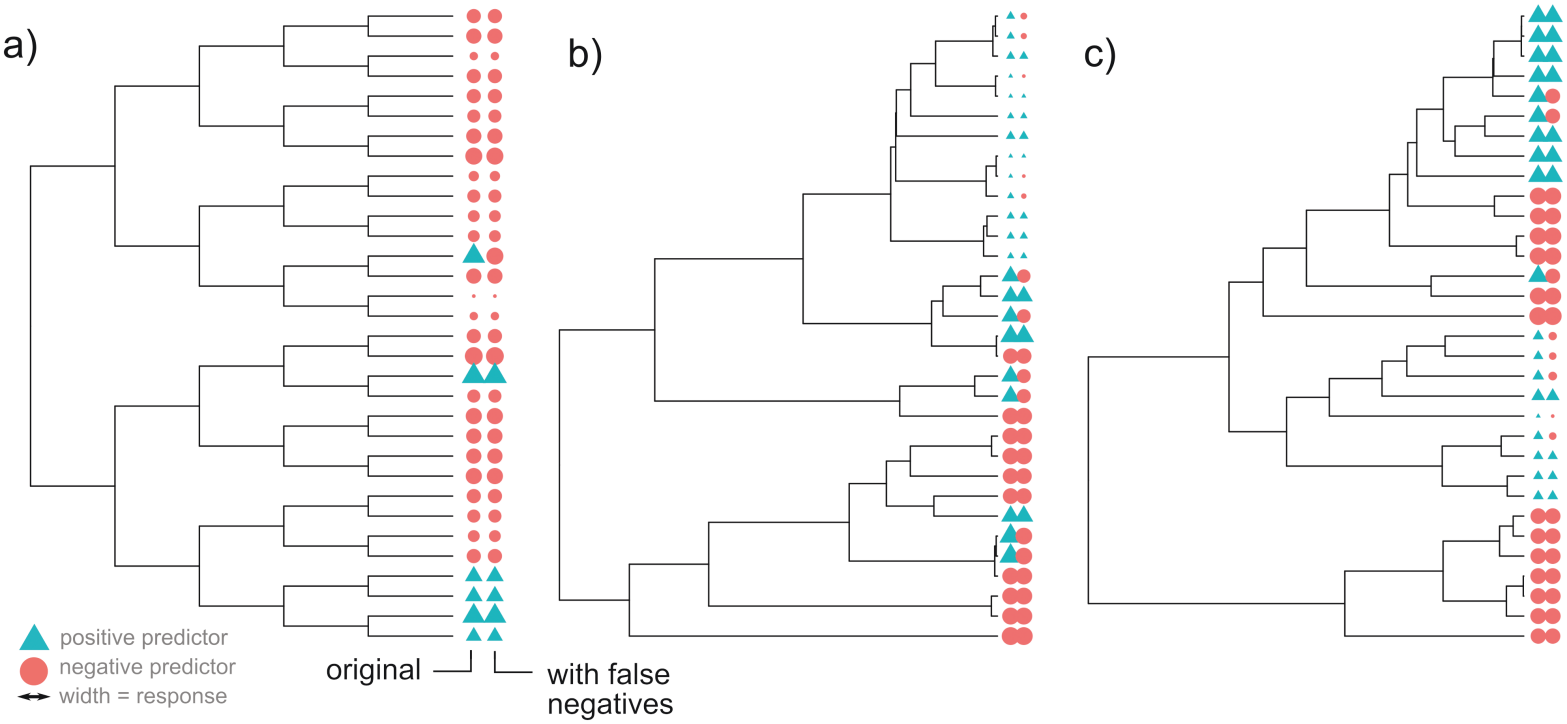
Example simulated phylogenies in the control studies. In each case, the tip labels give the true states (left) and observed states after false negative observations of the predictor (right). In this example, predictor evolution is irreversible, so that once the positive value is acquired it is never lost. Color gives predictor value (circles negative, triangles positive); the size of each symbol gives the response value. (A) A symmetric, balanced tree with no influence of predictor on response. (B and C) Birth–death trees with different death parameters, with a strong influence of predictor on response.


We built a series of synthetic models where predictor and response co-evolved on artificially constructed phylogenetic trees (Fig. 1). These trees either had uniform branch lengths or branch lengths determined by a simulated birth–death process with different parameterizations (Stadler 2011; Paradis and Schliep 2019). We then use a simple random model to generate values for a model ecological feature *X* and oDNA gene count *Y* on the phylogeny. The evolutionary rule for constructing descendant state (*X*_*d*_, *Y*_*d*_) from ancestor state (*X*_*a*_, *Y*_*a*_), given a branch of length *t*_*a,d*_ linking the two, was

$$\begin{array}{l} X_{d}=\;1-X_{a}\;with\;probability\;r;\;X_{a}\;with\;probability\;1-r \end{array}$$ (1)

$$\begin{array}{l} Y_{d}=Y_{a}-dY(X_{a},t_{a,d}), \end{array}$$ (2)

where


$$\begin{array}{l} dY(X_{a},t_{a,d})∼t_{a,d}U(0,m)\;if\;X_{a}=0;t_{a,d}U(c,m+c)\;otherwise. \end{array}$$ (3)

In other words, each ancestor-descendant step has a probability *r* of changing the descendant’s *X* value. The descendant’s *Y* value is inherited from its ancestor, with a random reduction *dY* of characteristic size *m* scaled by the branch length, and shifted to higher magnitudes if the ancestor’s X value is nonzero. Any negative *Y* values that emerged under Equations (1–3) were set to zero.

To imagine a particular biological example, parasitism (*X* = 1) evolves at random through the phylogeny, and increases the rate of with which ptDNA gene count *Y* is lost when it appears. We also considered irreversible changes in *X*, that is, replacing Equation (1) with


$$\begin{array}{l} X_{d}=1\;with\;probability\;r;\;X_{a}\;with\;probability\;1-r \end{array}$$ (4)

So that once *X* acquires value 1 (generally corresponding to the presence of an ecological feature) it is never lost in that lineage. We reasoned that irreversible dynamics would be an appropriate model when adaptation to a particular feature (perhaps, e.g., parasitism) make the subsequent loss of that feature highly unlikely.

The picture here is not that a given predictor value *X* imposes an optimal value of *Y* to which the system adapts (Hansen 1997)—this might not be so reasonable for our purposes, where multiple influences other than *X* influence *Y* (Giannakis et al. 2022; García-Pascual et al. 2023). Rather, our picture is that a given predictor value influences the rate at which species adapt towards lower values of *Y* (see “Discussion” section).

After simulating evolution, each tip on the phylogeny had a pair of values (*X*, *Y*). Those tips with *X* = 1 were assigned an “observed” value *X* = 0 with probability *p*. This false negative observation modeling is designed to mimic the false negatives which are known to be prevalent in several of our datasets, where records of organismal traits are sparse: a positive observation likely corresponds to a true positive (TP), but a negative observation could correspond to a true negative or an incomplete observation.

The parameters we varied were *r*, the probability of the predictor changing value along a given branch; *c*, the influence of the *X* = 1 case on the rate of *Y* evolution; and *p*, the probability with which a true *X* = 1 value led to an *X* = 0 observation. We also varied the size of the tree *n* and *ν*, the death rate parameter in the birth–death process that generated random phylogenies.

### Phylogenetic Generalized Least Squares and (Generalized) Linear Models

We used R packages *ape* (Paradis and Schliep 2019) and *nlme* (Pinheiro et al. 2020) to implement phylogenetic generalized least squares (PGLS) (Grafen 1989; Symonds and Blomberg 2014; Revell and Harmon 2022). Specifically, we used a range of evolutionary models to specify the form of the covariance matrix: a simple model assuming characters evolve with Brownian motion (Felsenstein 1985; Martins and Hansen 1997), Grafen’s method (1989), scaling covariances by Pagel’s *λ* (Pagel 1999; Freckleton et al. 2002), and the Martins–Hansen approach of exponentially decaying covariance over evolutionary time (1997). We used these different covariance structures to be as flexible as possible in our representation of the evolutionary dynamics of oDNA gene loss and its possibly changing rates over evolutionary time (Janouškovec et al. 2017). *P* values were provided based on a chi-squared null distribution (Susko 2003), which, in turn, assumes normality of residuals, which is generally not the case for our data. Hence, we test the conclusions from this analysis against the simulated systems above to assess the validity of this interpretation. As a numerical alternative, we also used the *phylolm* package (Tung Ho and Ané 2014) for phylogenetic linear models (PLM, expected to behave very similarly to the PGLS approach) and phylogenetic generalized linear models (PGLM, specifically using Poisson regression) with a Brownian-motion-derived covariance matrix. Numerically, *phylolm* uses generalized estimating equations to increase the computational efficiency of these regression processes (Paradis and Claude 2002). *P* values here again are dependent on some parametric assumptions, so we refer to tests against simulation for interpretation as in the text. We used both PGLS/PLM and PGLM—although our response variable is always a non-negative integer—as we did not a priori know the relative robustness of the two approaches to the various evolutionary assumption violations in our system. We reasoned that if PLM was less sensitive to breaking evolutionary assumptions then its statistical performance might still be competitive despite its unrestricted response variable structure. To summarize, we used *ape* and *nlme* for PGLS exploring different covariance structures, and *phylolm* using generalized estimating equations to implement PLM and Poisson PGLM with Brownian-motion covariance structure.

### Within-Family Comparisons

We also used a non-parameteric approach for comparing relatives, following the broad idea in (Maddison 2000). We first assign *X* and *Y* values to all internal nodes in the phylogeny. Predictor *X* is set to 1 for an ancestor if all its descendants have value 1, otherwise, the ancestor is set to 0. Response values *Y* for an ancestor are set to the mean of its descendants. We then identify sets of siblings (among internal nodes as well as tips) that differ in their *X* values. For each, we record *Y*^*−*^ and *Y*^*+*^, respectively the response value in the siblings with predictor value 0 and 1 (in the case of trees that are not fully resolved, where an ancestral node may have more than two descendants, we take the mean response value across siblings with the same predictor value). The list of (*Y*^*+*^ – *Y*^−^) values is recorded across all sets of siblings in the tree where diversity exists in the predictor value. This overall process is demonstrated in Supplementary Fig. 1 (Supplementary Information can be found online at the Zenodo repository: https://zenodo.org/doi/10.5281/zenodo.10693678). Given this list of differences, we use two approaches to test against the null hypothesis of a zero difference (siblings with predictor value 0, and those with predictor value 1, have no difference in response values). Approach (a) is a Wilcoxon signed-rank test (hence, a non-parametric matched-pairs test). Approach (b) uses bootstrap resampling, over the set of differences, to build a sampling distribution for the mean difference. We then use the percentile method to obtain the probability that zero (or a more extreme value) lies within this distribution.

### Accounting for Clade Influence

Much variability in oDNA gene count occurs at the level of supergroups and kingdoms (Janouškovec et al. 2017; Giannakis et al. 2022). In a parallel approach attempting to account for this, we also used several approaches to “block” different eukaryotic clades as sources of oDNA gene count variability and analyze the remaining variance. The picture here is not that of a phylogenetic “constraint” on evolution (Losos 2011), but rather that different deep-branching taxa may have different “baselines” of oDNA gene count, resulting from independent early evolutionary gene loss processes (Janouškovec et al. 2017). We wanted to know whether, if we account for these baseline differences between taxa, ecological effects predict oDNA variability across taxa. We picture these different baselines as the random intercepts in the mixed model and indeed are then interested in the fixed effects that correspond to relationships that hold across taxa after accounting for these baselines. To be as general as possible we also explored the case where slopes can also vary across taxa (reflecting, e.g., that some taxa are more permissive to ongoing gene transfer than others, and that loss rates may correspondingly vary).

We used linear mixed models and Poisson generalized linear mixed models (LMMs and GLMMs) with random effects on either intercepts alone, or both intercepts and slopes (selected via Akaike Information Criterion, AIC), associated with eukaryotic clade. We also took a non-parametric approach using the Scheirer–Ray–Hare (SRH) test, which resembles a generalization of the better-known Kruskal–Wallis test to allow for two factors and replicated observations (Scheirer et al. 1976). Specifically, the first assigned branch after the common eukaryotic ancestor from NCBI’s Common Tree was used as the blocking variable, and the feature of interest was used as the group variable. If a particular feature was only found to take different values in a single clade, the Kruskal–Wallis test was used within that single clade to provide an analogous result. The SRH test was implemented using the *rcompanion* package (Mangiafico 2020). Finally, we attempted to follow the philosophy of (Maddison and FitzJohn 2015), where we independently normalized the gene counts within a clade by subtracting that clade’s mean count from every observation, before analyzing with PLM as above.

### Curation of Organismal Properties from Databases

The trait dataset used in our analysis was assembled using a combination of automated and manual methods. Species names and genetic information (such as organellar genome size, GC content, and taxonomical information) were collected via NCBI’s Organelle database (O’Leary et al. 2016) and processed according to the pipeline in (Giannakis et al. 2022). This pipeline, in brief, involves curating the acquired set of annotated oDNA genome records—which, given their breadth, are often inconsistently labeled—by systematizing the annotation of genes across species and taxa. This curation is achieved both through manual clustering of gene labels, mapping nonstandard annotations to a standardized equivalent, and through an automatic all-against-all BLAST comparison and subsequent clustering and relabeling. After curation, we retained 9296 mtDNA sequences (8835 of which are metazoans) and 4264 ptDNA sequences. The majority of the ecological trait entries were acquired via Encyclopedia of Life (Parr et al. 2014) through individual queries for traits of interest and then cross-referenced with some domain-specific databases (like GloBI (Poelen et al.2014), but also others, see following references). Encyclopedia of Life is an open encyclopedia that provides a catalogue with plenty of traits and taxonomic information on organisms, which consists of contributions and curation by experts and organizations. Data on palm trees specifically were collected through (Kissling et al. 2019). Organismal habitats and traits related to cross-species interactions (including parasitism) were taken from (Cohen et al. 2020), a large ecological scale survey on species interactions. For the majority of plant data, ecological traits and characters were taken from Encyclopedia of Life and crosschecked with the TRY database (2018 update) (Kattge et al. 2020).

For the cross-eukaryote data, NCBI’s Common Taxonomy Tool was used to obtain a phylogenetic topology constructed from independently provided taxonomic information (Federhen 2012). More specific phylogenetic information was taken from the *U.PhyloMaker* R package (Jin and Qian 2022, 2023) which uses the Plant megaphylogeny found in Jin and Qian (2022) comprised of phylogenies from Smith and Brown (2018) and Zanne et al. (2014). The plant phylogeny assembled in Jin and Qian (2022) and sourced from Zanne et al. (2014) and Smith and Brown (2018) gave us a sufficient number of matches (up to the level of genus) to our list of species (120 for the species with mitochondrial gene count and 3851 for plastid) and we were able to test different scenario.

Besides the automated retrieval of trait values from the above resources via custom R and Python scripts, manual insertions and corrections took place over individual observations, either through parsing Wikipedia pages (see below) or via ongoing literature reading and personal communications. Data were processed and assembled into two major datasets (one for each organelle) using custom R scripts.

### Semi-Automated Curation of Organismal Properties from Less Systematized Sources

This approach assumes that an XML dump of articles (e.g., Wikipedia pages, PubMed abstracts, or NCBI entries) corresponding to a set of taxa of interest can be obtained (e.g., via Wikipedia’s Special:Export API (https://en.wikipedia.org/wiki/Special:Export). A custom Python script then searches for a given regular expression pattern in such an XML file, and produces an HTML file designed to assist manual parsing of all the entries matching that pattern. The HTML page consists of the blocks of text surrounding each pattern match, labeled by page name. Hyperlinks and other interesting text are accompanied by checkboxes, which when clicked populate a text box on the right-hand side of the page with the text immediately before the checkbox. At the bottom of the HTML page, there is a summary button that compiles all the text from other boxes into a single comma-separated list.

The user can quickly check any examples where a given element of text (e.g., a species or taxon name) genuinely corresponds to the feature of interest, then compile all these positive cases into a summary list. Regular expressions can be arbitrarily complex (e.g., to identify single-celled organisms we found*/[Uu]nicell|[Uu]ni-cell|[Ss]ingle-cell/* to give useful results).

### Software References

We used R (R Core Team and Team 2022) with packages *ape* (Paradis and Schliep 2019), *phytools* (Revell 2012), *phylolm* (Tung Ho and Ané 2014), and *phangorn* (Schliep 2011) for phylogenetic analysis, *taxizedb* (Chamberlain and Arendsee 2020) and *stringr* (Wickham 2019) for data curation, *lme4* (Bates et al. 2015), *nlme* (Pinheiro et al. 2020), and *rcompanion* (Mangiafico 2020) for statistical analysis, and *ggplot2* (Wickham 2011), *beeswarm* (Eklund 2016), *ggrepel* (Slowikowski 2021), *gridExtra* (Auguie and Antonov 2017), *ggtree* (Yu et al. 2017), and *ggtreeExtra* (Xu et al. 2021) for plotting. oDNA profiles were taken from the pipeline in Giannakis et al. (2022). All software and data for the phylogenetic analysis are available at https://github.com/StochasticBiology/comparative-odna, with a permanent link to the post-review version of the code via Zenodo at https://zenodo.org/doi/10.5281/zenodo.10693678.

## Results

### Sensitivity and Specificity of Comparative Phylogenetic Methods With Organelle-Like Evolution and Varying Data Properties

We sought to understand the impact of the above issues—sparse, heterogeneous, uncertain observations and reductive evolutionary dynamics—on the performance of comparative approaches seeking correlations between predictor and response. We simulated a range of synthetic evolutionary systems on different tree topologies, involving a response variable decreasing at a rate that may depend on an (ecological) predictor variable that may itself change state over evolutionary time. Observations of the predictor variable were occluded with a false negative probability (see “Methods” section; Fig. 1). This setup is designed to match the complications in organelle genome analysis.

Because of its flexibility, we began with PGLS, with correlation estimates assuming that traits evolve under Brownian motion, to estimate species correlations (Grafen 1989; Symonds and Blomberg 2014). We also explored other approaches for investigating correlations while accounting, or not, for phylogenetic coupling (“Methods” section; Supplementary Figs. 2 and 3). We explored the use of different evolutionary models to estimate the variance–covariance matrix (capturing common ancestry in the observed data, and hence describing covariance between residuals in these regression models (Blomberg et al. 2012)) as well as the naïve case with no accounting for phylogenetic relationships. In parallel with a classic numerical implementation of PGLS, we tested a set of numerical implementation using generalized estimating equations for phylogenetic linear modeling (PLM; expected to behave very similarly to PGLS) also using the Brownian evolution model (Tung Ho and Ané 2014), and phylogenetic generalized linear modeling (PGLM) with a Poisson response distribution (Paradis and Claude 2002; Tung Ho and Ané 2014). We also used a nonparametric pipeline based on comparisons of related species or clades with different values of the predictor without invoking a particular evolutionary model, following the principle of Maddison (2000)—where comparisons are only made between pairs of clades that are phylogenetically separate given the tree structure. Finally, we explored the effects of considering or neglecting branch length information with different evolutionary models for covariance structure. We assessed the specificity and sensitivity of these methods as tree size, tree topology, false negative observation probability, magnitude of predictor–response relationship, and prevalence of positive predictor values were varied.

**Figure 2.**
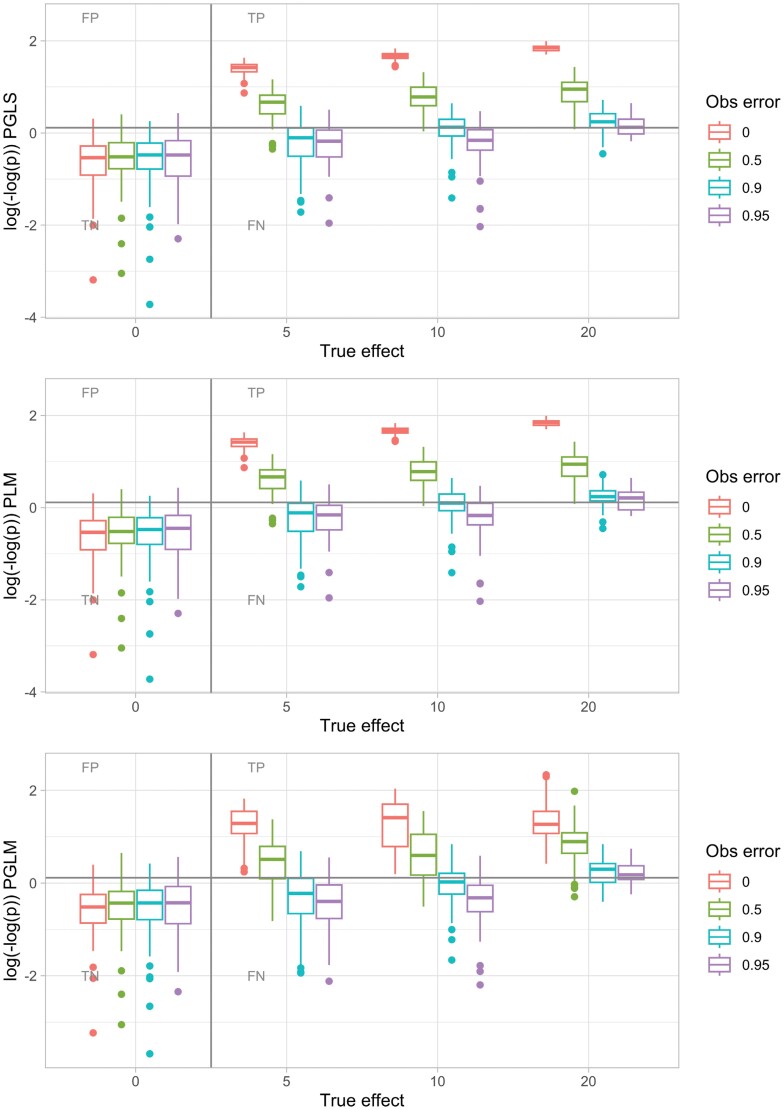
Example of PGLS, PLM, and PGLM sensitivity–specificity investigation. Evolutionary dynamics were simulated on a tree with 256 leaves, with differing true effect *c* linking predictor value and response evolution. Predictor value was allowed to change reversibly through evolution; the equivalent plot for irreversible dynamics is shown in Supplementary Fig. 4. Observations of the predictor value were occluded with an observation error parameter giving the probability that a positive value is observed as a negative. The gray line corresponds to *P* = 0.05; points above would be interpreted as the presence of a signal (without multiple hypothesis correction), and points below as the absence of a signal. PGLS with covariance structure derived from a Brownian model rarely gives FP correlations and has substantial power to detect TP correlations even for high observation error probabilities; PLM is almost identical in performance. PGLM likewise limits FP and retains power to detect TP, although the spread of *P* values reported by PGLM for positive cases is rather broader. *nlme* (Pinheiro et al. 2020) was used for PGLS; *phylolm* (Tung Ho and Ané 2014) for PLM and PGLM with generalized estimating equations. Here, an average of 8 evolutionary events innovated a positive predictor value; the effects of other simulation parameters and methods are shown in Supplementary Figs. 2–6.

The approach neglecting phylogenetic structure and treating each species as an independent sample led to, as expected, a substantial FP rate (as observations that share similarities with a common ancestor are effectively treated as independent). The nonparametric approach performed poorly, with low statistical power, although FPs were limited. PGLS and PGLM, on the other hand, performed well at detecting extant links between variables, even with substantial departures from the idealized case above (Supplementary Figs. 2 and 6). An example is shown in Fig. 2. Not unexpectedly, the PLM approach behaved almost identically to the PGLS approach (Fig. 2, Supplementary Figs. 2 and 3).

Our interpretation of this perhaps surprisingly good performance for these parametric approaches is that although the evolutionary dynamics in our system are rather different from the ideal case (e.g., continuous traits evolving according to Brownian motion), the latter still suffices to provide a reasonable estimate of the expected correlations between different species. Although the residual structure from our GLS model fit is not (cannot be) perfectly normal, the approach is general enough to allow informative follow-up analysis. The one-way occlusion of observations (positives may be observed as negative, but not the other way round) means that signal is preserved more than a completely random observation setup would allow.

We observed similar performance across methods for the cases of reversible and irreversible predictor variable evolution (Equation (1) vs. Equation (4); Fig. 2 vs. Supplementary Fig. 4; Supplementary Figs. 2 and 3). Where there was a difference, the comparative approaches typically performed marginally better in the case of reversible evolution. This improvement is likely due to the increased diversity of predictor-response pairs that will typically arise when reversibility is supported. The potential confounding effect in the reversible case—where a lineage evolves under one predictor value for most of its history, then switches to another value before observation—does not seem to provide a strong enough negative influence on performance to overcome this increased diversity.

One disadvantage of the classic PGLS approach is its relative computational intensity. For phylogenies of the size involved with our organelle data (over 9000 mtDNA sequences and 4000 ptDNA sequences), the construction and use of an *N* × *N* covariance matrix (Martins and Hansen 1997) become quite a computational challenge (taking several core days to analyze dozens of predictors). The generalized estimating equations approach for PLM (and PGLM) (Paradis and Claude 2002; Tung Ho and Ané 2014) is much faster (taking only core seconds for the same analysis), as is the relative-comparison approach of recursively labeling a tree then seeking diverse siblings.

### Semi-Automated Pipeline for Labeling Organismal Traits from Unsystematized Data Sources

Encouraged by the potential of this comparative approach to detect correlations in the face of awkward and imperfectly observed data, we next attempted to gather information on organismal traits that could be linked to oDNA gene retention. Many of our traits of interest are not systematically assigned across a full list of eukaryotic species in a dedicated database (see “Methods” section). However, less formalized online sources contain a substantial amount of information about some traits. Wikipedia (https://www.wikipedia.org/), for example, contains articles about many taxonomic groups and individual species, which often describe habitats, free-living versus parasitic lifestyles, multicellular versus unicellular physiology, and so on. Unfortunately, automated methods to extract this information are not (to our knowledge) available, and are challenging to construct. The huge variety of phrasings of this information and difficulties in automatically resolving ambiguous text suggested that a manual curation would be necessary. But reading (for example) the Wikipedia page for thousands of entries would be a tremendous investment of time.

We briefly explored using ChatGPT (https://chat.openai.com/chat) to gather organismal traits. This was unsuccessful. ChatGPT readily fabricated information about traits in both taxonomic groups and individual species. It was unable to either reliably assign traits to entities or to produce a list of entities with a given trait.

**Figure 3.**
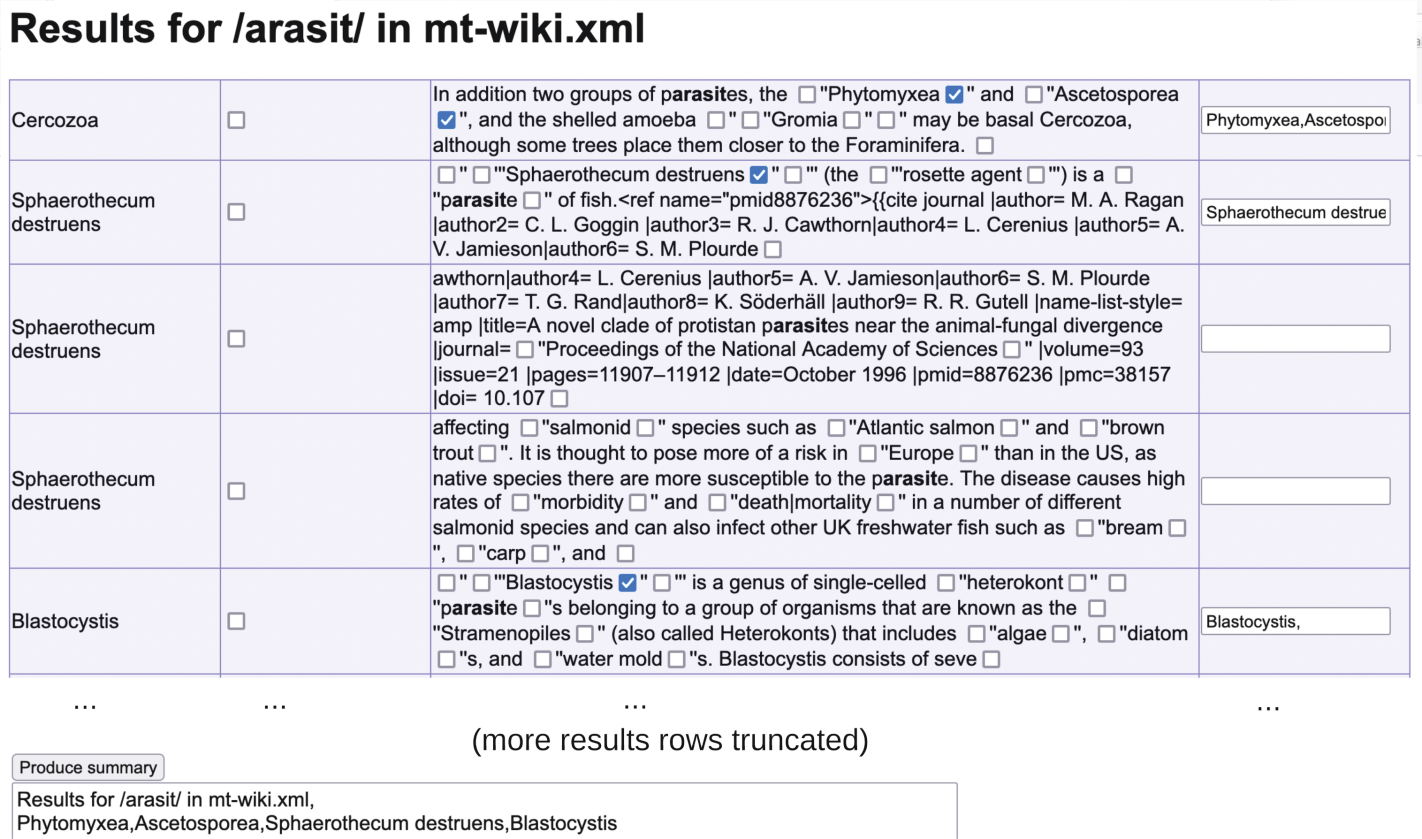
Screenshot of user interface for semi-automated extraction of organismal traits from online encyclopedia content. A custom script seeks a regular expression associated with a trait (here*/arasit/*, designed to match [Pp]arasit[ic/ism/etc]) in the corpus of Wikipedia articles describing species and taxa within our phylogeny of interest. The text surrounding each instance of the expression is reported, with check boxes allowing the selection of hyperlinked terms that are manually deemed to match the trait (some examples demonstrated)—which are then stored in boxes on the right, where terms can also be manually entered. An example set of entries is shown in the figure; this query returned several hundred more. After parsing entries (many truncated here), a summary button creates a comma-separated list of all positively identified or entered terms.

To make progress, we constructed a pipeline to semi-automate the process of gathering information on a given trait from a source like Wikipedia (“Methods” section). Illustrated in Figure 3, this pipeline allows a user to provide a list of species and taxa to query, then returns every instance in the data source where an entry associated with elements of that list contains a term of interest. This set can either be immediately used or, as shown in Figure 3, manually parsed to ensure reported entries genuinely correspond to the term of interest. We used this pipeline to gather information on several traits for our oDNA datasets: parasitism, extremophilia, multicellularity, sessility, flagellated physiology, and different photosynthetic modes, but we anticipate that its use could be much more general.

### Features Correlated with Mitochondrial and Chloroplast Gene Counts Across Eukaryotes

**Figure 4.**
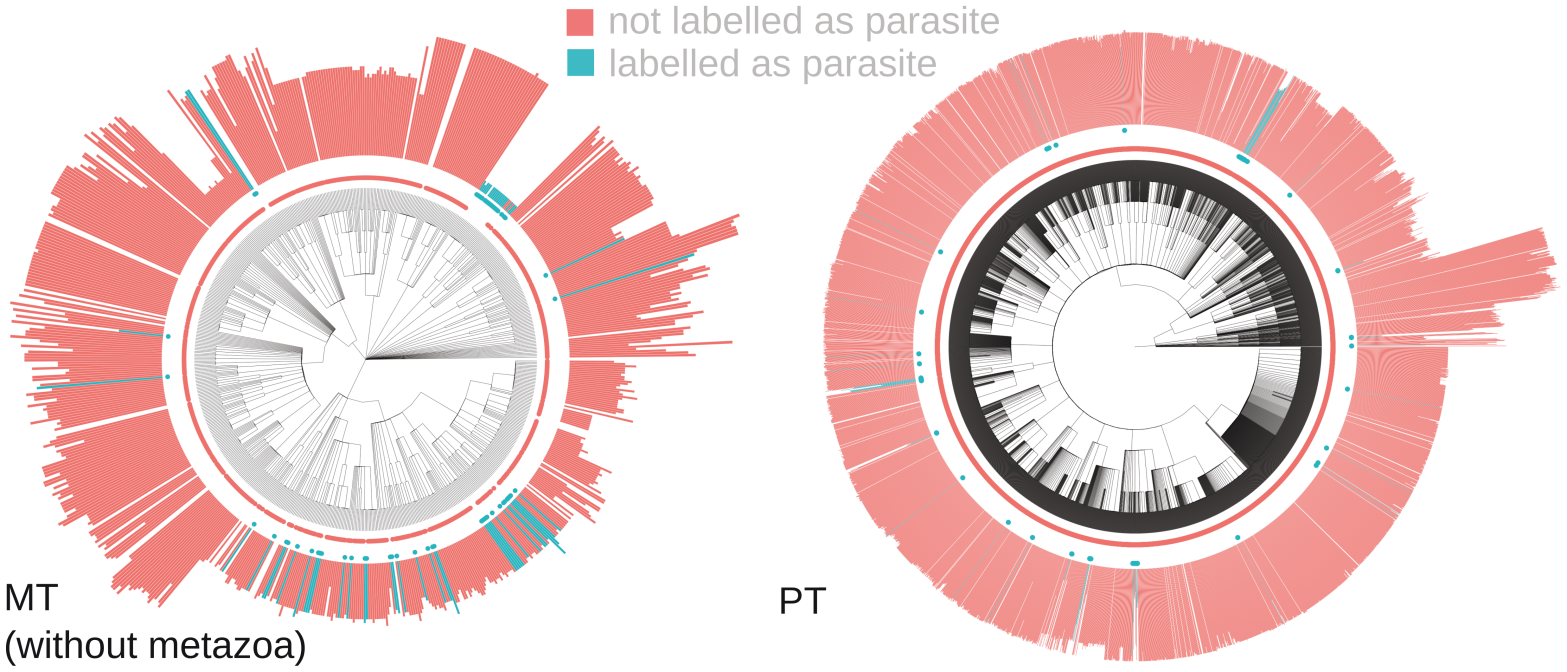
Example oDNA data. (left) mtDNA, without metazoa for clarity; (right) ptDNA. Colour (and central point markers) give the predictor value (parasitism positive or negative). The length of bars gives oDNA gene count.

Combining the above control studies, comparative workflow, and data-gathering pipelines, we were in a position to explore the scientific question of which organismal traits are correlated with organelle gene counts. We gathered organelle protein-coding gene counts from the automated analysis in Giannakis et al. (2022), ORF counts from NCBI RefSeq (O’Leary et al. 2016), and organismal properties from the data sources above, comprising 67 ecological and organismal features (“Methods” section; Supplementary Tables 1 and 2). We used estimated taxonomic trees from NCBI’s Common Taxonomy Tree Tool (Federhen 2012). For each property, we considered each possible value in turn as the “positive” case (an illustration, for the parasitism trait, is shown in Fig. 4). The dataset showed a strong phylogenetic signal, with Pagel’s *λ* exceeding 0.999 for both organelle counts.

Several issues immediately arose in the comparative analysis of these real datasets. While our benchmarking approach tested the effect of traits not being measured for all individuals, in the ecological data, not all traits are even defined for all eukaryotes. Initial empirical investigation suggested, for example, a link between the feature “palm” and mtDNA gene count. But further investigation quickly showed that this was simply reporting the fact that palms are plants, and animals (which dominate the dataset) are not. In this and many other cases, the predictor can be regarded not just as negative, but undefined, for a subset of species. In these cases, we performed analysis only on the subtree rooted at the common ancestor of all positive-labeled species (thus restricting the “palm” analysis to plants). We used both PLM with a Brownian-motion derived correlation structure (reasoning that the strong agreement between PLM and PGLS results justified the choice of the much faster approach) and PGLM to explore links between the (predictor) property and the (gene count) response.

The next issue was one of (outlier) influence. In several cases, a significant correlation was reported by the pipeline but was completely dependent on a single observation. An example is given in Supplementary Fig. 8, where the “effect” arises from a shifted ratio of *n* = 12 to *n* = 13 genes in Metazoa, but the ratio in the positive case is highly dependent on a single *n* = 12 observation. To avoid dubious cases like this, we filtered results: if either predictor value had only two associated response values, we required at least 6 observations of all response values to retain a result. This threshold was chosen through empirical investigation as a suitable guard against these small-number effects without leading to many ecological features being discarded.

**Figure 5.**
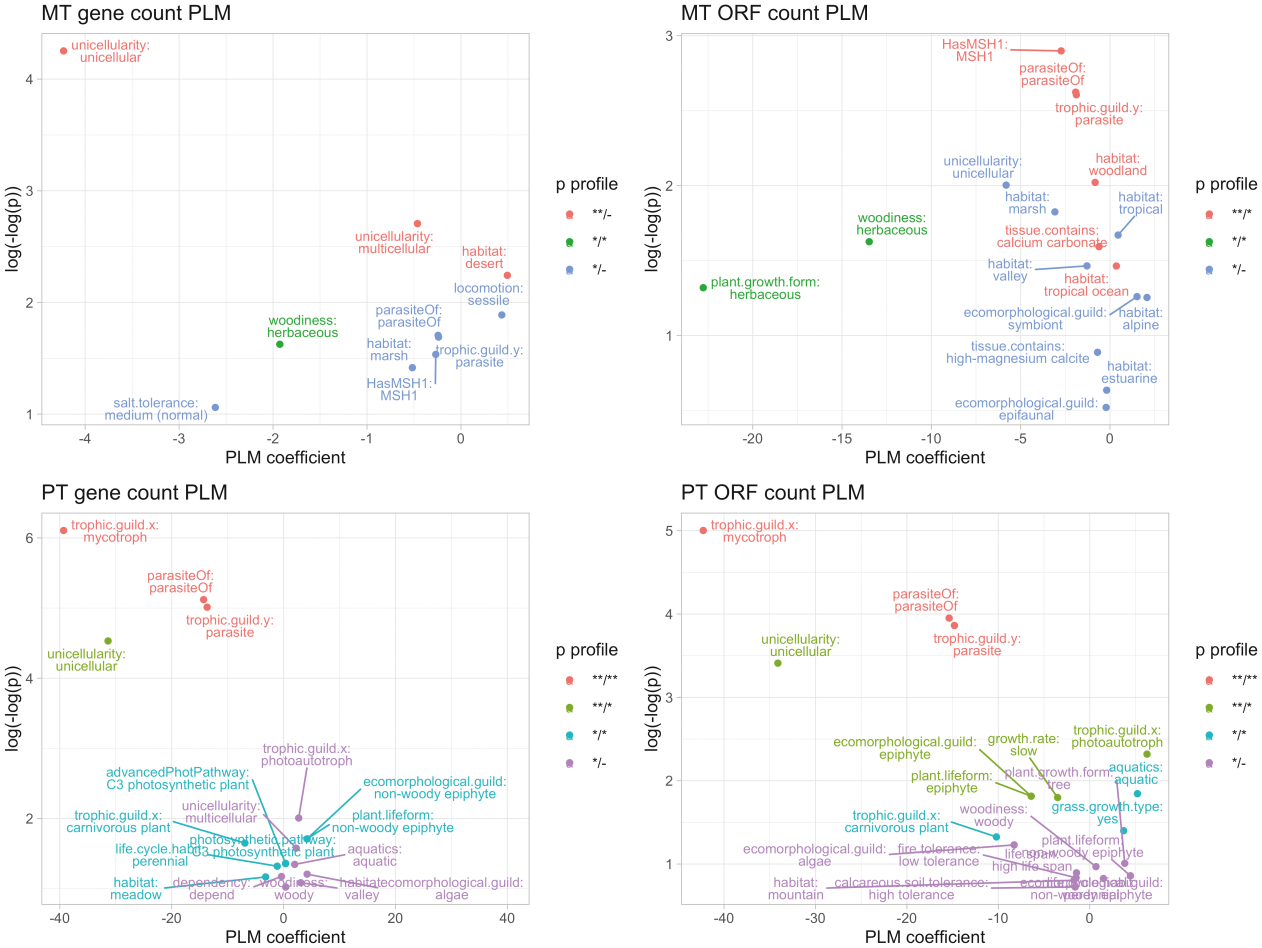
Features correlated with oDNA gene counts. PLM coefficients (*x*-axis) and *P* values (*y*-axis, double-logged and inverted) for relationships between different organismal traits and organelle DNA gene counts (mtDNA and ptDNA), counted as confirmed protein-coding genes or CDS regions. This analysis is applied to the cross-eukaryote dataset as described in the text. The figure shows statistics from the PLM approach (the corresponding PGLM statistics are shown in Supplementary Fig. 9), but colors correspond to profiles of statistical significance using both PLM and PGLM approaches. ** denotes *P* < 0.05 after Bonferroni; * *P* < 0.05 without correction; –*P* > 0.05 (e.g., **/* means one approach gave a Bonferroni-robust *P* < 0.05 and the other gave 0.05 not robust to Bonferroni). The PLM coefficient gives the average inferred change in gene count if an organism has a given property. The majority of traits give substantially higher *P* values and lower-magnitude coefficients; plots are vertically truncated to focus on the more robust results. Example of the full distributions of oDNA gene counts with different features can be seen in (Supplementary Figs. 13–14).

Finally, we found some issues in the original dataset, including, for example, metazoans being assigned plant traits. We identified and fixed these issues manually. The results for predictors of confirmed protein-coding gene counts, and CDS region counts, are shown in Fig. 5 and summarized in Table 1. We report both results that are robust to Bonferroni correction respecting all the predictors tested, and those that display *P* < 0.05 but are not robust to Bonferroni correction (which could be viewed as somewhat conservative, especially given the large number of predictors considered).

**Table 1.** Summary of identified predictors of oDNA gene counts.

Feature	Clade	Direction	Gene count *P* value profile	ORF count *P* value profile
**mtDNA**				
Parasitism	Eukaryota	–	*/–	**/*
Herbaceous growth form	Streptophyta	–	*/*	*/*
Woodland habitat	Metazoa	–	–/–	**/*
Tropical ocean habitat	Metazoa	+	–/–	**/*
Has *msh1*	Eukaryota	–	*/–	**/*
CaCO_3_ tissue	Metazoa	+	–/–	**/*
Unicellularity/multicellularity†	Eukaryota	–	**/–	*/–
Desert habitat	Eukaryota	+	**/–	–/–
**ptDNA**				
Parasitism	Eukaryota	–	**/**	**/**
Mycotroph	Magnoliopsida	–	**/**	**/**
Unicellularity	Eukaryota	–	**/*	**/*
Epiphyte††	Streptophyta	–	–/–	**/*
Photoautotroph	Eukaryota	+	*/–	**/*
Slow growth rate	Streptophyta	–	–/–	**/*
Carnivorous plant	Magnoliopsida	–	*/*	*/*
Aquatic	Eukaryota	+	*/–	*/*

Direction gives the sign of the coefficient of a predictor on gene count response. As in Figure 5, *P* value profiles describe statistical significance in PLM and PGLM approaches: ** denotes *P* < 0.05 after Bonferroni; * *P* < 0.05 without correction; − *P* > 0.05. For example, parasitism is correlated with mtDNA gene count at the *P* < 0.05 level by one of PGLM or PLM (*/–), and with mtDNA ORF count at the Bonferroni *P* < 0.05 level by one approach and the *P* < 0.05 level by the other (**/*). † Both unicellularity and multicellularity appear with gene count effects in the same direction—reflecting a probably artefactual comparison with the unlabeled subset of the data. †† Non-woody epiphytes appear as a significant correlate, but with the opposite sign, in the gene count dataset, suggesting an interaction between woodiness and epiphyte traits.

We also conducted the same analysis for two alternative phylogenies, in an attempt to further improve robustness. In case the oversampling of metazoan mtDNA retained an influence on results from other taxa, we also removed metazoans from the mtDNA data and performed the same analysis (Supplementary Fig. 10); most of the non-metazoan traits in Fig. 5 and Table 1 were retained, and an additional significant link with a tropical habitat (associated with higher mtDNA gene counts) was observed. Finally, we also performed the analysis with a smaller, plants-only phylogeny with complete branch length estimates (also removing these estimates to further test the influence of branch length information), in Supplementary Figs. 11 and 12. Here, the ptDNA correlates were very comparable to those from the cross-eukaryotic data, but the mtDNA correlates differed. Desert habitat and herbaceous plant form were retained, but several features relating to environmental tolerance (e.g., soil and shade) also appeared. The results from neglecting branch lengths in this plant dataset were largely comparable to those retaining branch length estimates but with less statistical power (typically less robust *P* values).

Our approaches blocking, or assigning random effects to, eukaryotic clade and analyzing residual variance (see “Methods” section) broadly agreed with these features. For the non-parameteric approach blocking clade (Supplementary Figs. 13–14), the set of suggested predictor factors at the Bonferroni *P* < 0.05 level for mtDNA including traits related to parasitism, unicellularity, plant lifeform, and animal motility. For ptDNA the traits were parasitism, plant growth form and lifeform, carnivory and mycotrophy, unicellularity, and leaf morphology. The mixed-model approaches assigning random effects to clade, and the approach where gene counts were normalized by subtracting the clade’s mean count, produced broadly similar results to Fig. 5 (Supplementary Figs. 15–16). Here, parasitism and unicellularity appear once more for mtDNA gene counts, with herbaceous plant form, sessility, bryophytic lifeform, and forest environments also appearing. Some further features identified by the mixed-model approach alone include C_3_ photosynthesis and broad leaf morphology (both associated with lower mtDNA gene counts) among Streptophyta. For ptDNA, the mixed modeling approach once more identified parasitism, mycotrophy, carnivory, and unicellularity, with some further features including a meadow habitat and perennial life cycle (both associated with lower ptDNA gene counts). It must be repeated that these approaches only account for relatedness at the highest level of the eukaryotic tree, but do attempt to directly control for the sometimes dramatic systematic differences in oDNA gene counts across different clades.

## Discussion

For both mitochondria and chloroplasts, we anticipated one known link: parasitic species often retain fewer organelle DNA genes. However, particularly in the case of mtDNA, it was not clear how strong the associated signal would be: many parasitic animals exist, for example, that have the same mtDNA gene profile as free-living animals. Many deep-branching taxa, including Apicomplexans, are parasitic and have highly reduced (or no) mtDNA—but such examples may reflect similarity by descent and, in fact, provide little support for a general relationship across life (Fig. 4). However, parasitism was readily detected by the pipeline (Fig. 5, Table 1), and in the ptDNA case, several other features related to a reduced dependence on photosynthesis (mycotrophy, carnivory) clearly emerged as predictors of reduced ptDNA counts.

It has been hypothesized that organelle gene retention may in part depend on features of an organism’s ecology and environment (Johnston 2019; García-Pascual et al. 2023); an instance of the principle of colocalization for redox regulation (Allen 2015; Allen and Martin 2016). The picture here is that if an environment imposes strong, varying demands on an organism’s metabolism and bioenergetic budget, the retention of oDNA genes may help organelles rapidly and individually respond to these challenges. By contrast, organisms in more stable, less challenging environments can allow organelle genes to transfer to the nucleus, as rapid response is less important and the nucleus provides a genetically preferable environment.

Several of the ecological traits suggested by this analysis support this picture. ptDNA counts are lower in organisms that are less exposed to photosynthetic demands: parasites and those species with other nutrient and energy sources (mycotrophs, carnivorous plants). Aquatic phototrophs and algae (a weaker signal for the latter set), both of which exist in dynamic aquatic environments, retain more ptDNA genes (Keeling 2010; Mohanta et al. 2020; Giannakis et al. 2022; García-Pascual et al. 2023). Parasites retain fewer mtDNA genes (even after accounting for phylogenetic links) (Hjort et al. 2010), and sessile organisms—which cannot move to evade environmental changes and challenges (Johnston 2019)—retain more. In mtDNA, there are some habitat-specific signals that also correspond with this picture: desert and tropical ocean habitats (with strong diurnal variability in temperature) are correlated with higher mtDNA gene counts, while woodland habitats (in a sense more buffered and stable) are correlated with lower ones. Weaker signals, only detected in a subset of analyses, also appear: alpine and tropical environments correlate with higher gene counts.

Other identified links fall less clearly into this broad picture and constitute potentially interesting lines for further investigation. Herbaceous plants (vascular plants with no woody stems) and epiphytes (plants that live on other plants but which do not derive nutrients from them) have lower mtDNA and ptDNA counts, respectively; metazoans with CaCO_3_ tissues seem to have a skew toward higher gene counts (inasmuch as diversity exists in metazoan mtDNA). We can very cautiously speculate a little on possible connections here. Herbaceous plants typically have shorter lifespans than woody ones, meaning that they may be exposed to comparatively little environmental change over their individual lives—potentially shifting the balance away from oDNA gene retention. Epiphytes, often protected by forest canopy and colonizing environmental niches that few other species compete for, may broadly experience more stable environments than free-standing species, again shifting the balance away from oDNA gene retention. The other signals identified by individual methods, including other habitats and particularly perennial life cycles, may be interesting targets for further investigation of other mechanisms, perhaps analogous to elevated organelle gene transfer in plants with selfing or clonal reproductive behavior (Brandvain, Barker, and Wade 2007; Brandvain and Wade 2009).

It is worth mentioning one absent result. Previous theory (García-Pascual et al. 2023) discussed the particular case of organisms in intertidal environments—exposed to strong, regular oscillations of temperature, water, salt, and more—as a subset of the general idea above linking dynamic environments to oDNA retention. That intertidal organisms may experience strong pressure to retain oDNA genes (and maintain their integrity (Edwards et al. 2021)) is qualitatively observed in, for example, plastid gene counts in seaweeds (Keeling 2010; Mohanta et al. 2020; García-Pascual et al. 2023). The analysis here did not highlight intertidal habitat as a strong predictor of oDNA gene count after accounting for phylogenetic correlations, although other habitat conditions (including desert and tropical ocean) subject to strong variability were detected. Of course, given the sparse sampling in our source data, an absence of a positive result cannot directly be interpreted as a negative one. Particularly given that we do see, as above, a signal associated with aquatic phototrophs and algae, it may just be that the specific “intertidal” label in our dataset is too sparse (only 6 organisms have this label in our plastid dataset, Supplementary Table 2) to have sufficient statistical power. Further, a detailed investigation with a specific experimental design focused on particular environments—rather than the broad approach deliberately adopted here—will provide future refinement of this picture.

These oDNA links must certainly be interpreted with some caution, for both scientific and technical reasons. Beginning with the scientific, Janouškovec et al. (2017) have demonstrated that oDNA gene patterns emerged rather early in many taxa, with ongoing evolution of gene content slowing (to the point of stalling in many animals and fungi) over time. Hypotheses linking oDNA profiles to environmental traits should, therefore, consider the environments faced by ancestral organisms while this process was ongoing, as well as those faced by present-day organisms that survive with these established gene profiles.

This evolutionary history also raises the question of how to construct the most appropriate synthetic model to compare different methods. Existing theory on oDNA evolution (García-Pascual et al. 2023)—based on mathematical modeling and by no means confirmed to be true—implicitly pictures stabilizing selection favoring a value that depends on the interplay between many predictors, including those determined by ecology. For continuous, reversible, univariate processes, such stabilizing selection is often captured with an Ornstein–Uhlenbeck model (Hansen 1997; Hansen and Bartoszek 2012). But organelle gene loss dynamics are discrete, often irreversible, and the rate of loss varies both over evolutionary history and between taxa (Janouškovec et al. 2017; Giannakis et al. 2022). We have, therefore, instead used a model where ecology influences the rate, not (directly) the “target,” of the evolutionary process. Within a particular clade, a model more directly reflecting stabilizing selection may be more appropriate, and this will be the target of future research.

The diversity in both patterns and rates of oDNA evolution across clades also raises the question of how best to account for phylogenetic influence in such taxonomically broad cases. Ongoing discussion exists in the literature about how, if at all, to account for hierarchical patterns of dependency in phylogenetic data. Early studies like (Nee et al. 1991) observed an example of Simpson’s paradox: a relationship between two variables in one direction in an amalgamated dataset, but in the opposite direction when the dataset was conditioned on another variable (clade, in that study). This behavior has been used to argue for the essentiality of accounting for phylogenetic influence; but it has also been argued that accounting for conditional dependencies between variables in data of this type can be a more powerful approach (Uyeda et al. 2018). With more complete data on ecological predictors, exploring the conditional dependencies between variables in this application would be an interesting and complementary route of investigation.

Of course, despite our efforts to preserve specificity and sensitivity, there are several more technical reasons why some of the effects we see may be statistical artifacts. If observations are not missing completely at random but are sampled in some systematic way connected to the relationship between predictor and response, the correlations we detect will be affected. While the comparative approaches we use attempt to account for the relatedness of samples, the high weighting given to metazoa in the mtDNA dataset may lead to some skewing of results if this accounting is imperfect. As the rates of oDNA evolution differ in different clades, single-valued descriptions of correlations between species may not capture all the nuances of behavior across the eukaryotic tree (though our synthetic control studies also have these differences between rates). Furthermore, correlation is not causation, and even if correlations do exist between these variables across eukaryotes, such correlations can only ever be indicative of interesting research directions, not definitive proof of mechanistic relationships. Further work exploring the new links we have observed here in more biological detail will be required for a full understanding of their evolutionary relevance. We hope, however, that our analysis has signposted some interesting avenues for further inquiry in the specific field of organelle genome evolution.

We further hope that this work has helped to illustrate some points of more general interest across systematics and phylogenetic comparative methods. We have, through simulation and comparison of methods, explored the general potential of comparative approaches to identify correlations when many traditional assumptions are not met and when data is (rather) unreliably observed. We have found that the comparative methods we analyzed are rather robust, both to departures from “idealized” evolutionary dynamics of the variables involved, and to observation noise. The latter point was of particular interest to us—in our synthetic studies, even when noise occluded a majority of positive observations of our predictor variables, correlations even of small magnitude were still detected by PLM and PGLM. This message is encouraging for the comparative study of data where variables are unreliably observed or not observed at all, suggesting that information on even a limited subset of species can help identify links between variables of interest. Such cases of limited information are likely to be common when studying taxonomically broad samples (as in our example of “all eukaryotes with whole organelle DNA sequences”), and we hope that our work here has demonstrated the power of these comparative methods (appropriately tested through simulation) in working with sparse and otherwise challenging data.

## Supplementary material

Data available from the Zenodo Digital Repository: https://zenodo.org/doi/10.5281/zenodo.10693678.

## Data Availability

Supplementary material, including data files and/or online-only appendices, can be found on Github https://github.com/StochasticBiology/comparative-odna and hosted in the Zenodo data repository: https://zenodo.org/doi/10.5281/zenodo.10693678.
